# Acid ceramidase is a novel drug target for pediatric brain tumors

**DOI:** 10.18632/oncotarget.15800

**Published:** 2017-03-01

**Authors:** Ninh B. Doan, Ha S Nguyen, Andrew Montoure, Mona M. Al-Gizawiy, Wade M. Mueller, Shekar Kurpad, Scott D. Rand, Jennifer M. Connelly, Christopher R. Chitambar, Kathleen M. Schmainda, Shama P. Mirza

**Affiliations:** ^1^ Biotechnology and Bioengineering Center, Medical College of Wisconsin, Milwaukee, Wisconsin, 53226, USA; ^2^ Department of Neurosurgery, Medical College of Wisconsin, Milwaukee, Wisconsin, 53226, USA; ^3^ Department of Radiology, Medical College of Wisconsin, Milwaukee, Wisconsin, 53226, USA; ^4^ Department of Neurology, Medical College of Wisconsin, Milwaukee, Wisconsin, 53226, USA; ^5^ Department of Medicine, Hematology/Oncology, Medical College of Wisconsin, Milwaukee, Wisconsin, 53226, USA; ^6^ Department of Biophysics, Medical College of Wisconsin, Milwaukee, Wisconsin, 53226, USA; ^7^ Obstetrics and Gynecology, Medical College of Wisconsin, Milwaukee, Wisconsin, 53226, USA; ^8^ Department of Chemistry and Biochemistry, University of Wisconsin, Milwaukee, Wisconsin, 53211, USA

**Keywords:** pediatric glioblastoma, acid ceramidase inhibitors, carmofur, glioblastoma, medulloblastoma

## Abstract

Pediatric brain tumors are the most common solid tumors in children and are also a leading culprit of cancer-related fatalities in children. Pediatric brain tumors remain hard to treat. In this study, we demonstrated that medulloblastoma, pediatric glioblastoma, and atypical teratoid rhabdoid tumors express significant levels of acid ceramidase, where levels are highest in the radioresistant tumors, suggesting that acid ceramidase may confer radioresistance. More importantly, we also showed that acid ceramidase inhibitors are highly effective at targeting these pediatric brain tumors with low IC_50_ values (4.6–50 μM). This data suggests acid ceramidase as a novel drug target for adjuvant pediatric brain tumor therapies. Of these acid ceramidase inhibitors, carmofur has seen clinical use in Japan since 1981 for colorectal cancers and is a promising drug to undergo further animal studies and subsequently a clinical trial as a treatment for pediatric patients with brain tumors.

## INTRODUCTION

Malignant brain tumors are the most frequent solid tumors in the pediatric population; they constitute 20% to 30% of all pediatric cancers and represent the predominant cause of cancer-related deaths in childhood [1]. In this study, we focus on three malignant brain tumors – atypical rhabdoid/teratoid tumor (ATRT), glioblastoma, and medulloblastoma. In particular, ATRT is associated with a miserable prognosis, with medial survival at 9 to 17 months [2]. No guidelines exist for optimal treatment and different multimodal protocols are presently being studied to improve outcomes [2]. For pediatric glioblastoma, most institutions employ surgery and adjuvant radiotherapy, but the role of chemotherapy remains investigational [3, 4]. Median overall survival ranges from 15 months up to 40 months in various small series [4]. Of the three, medulloblastoma has been the most studied with the best overall survival. The standard treatment is chemotherapy (adjuvant lomustine, vincristine, cyclophosphamide, and cisplatin) after reduced-dose radiotherapy concomitantly with vincristine [1, 5–7]. Reported 5-year event-free survival is 80% [6, 7]. However, given the longer life expectancy, a key concern is the development of long-term toxicity associated with current treatment protocols [6, 8, 9].

A potential new chemotherapeutic target for these tumors is acid ceramidase. The enzyme helps regulate activity of ceramides in cells, affecting survival, growth, and death of tumor cells [10]. High levels of this enzyme have been discovered in various types of human cancer [10]. Moreover, over-expression of the enzyme in tumor cells confers resistance to apoptosis, while inhibition augments response to chemotherapeutic agents and radiation [10]. In this study, we showed that acid ceramidase inhibitors are highly effective at targeting medulloblastoma, pediatric glioblastoma, and atypical teratoid rhabdoid tumors. The data suggest acid ceramidase is a novel drug target for pediatric brain tumors. We also demonstrated that an acid ceramidase inhibitor called carmofur, which has seen clinical use in Japan since 1981 for colorectal cancers, is a promising drug to undergo further animal studies and subsequently a clinical trial for pediatric patients with brain tumors.

## RESULTS

### Patient derived pediatric brain tumor cells express significant levels of ASAH1

**Figure 1:**
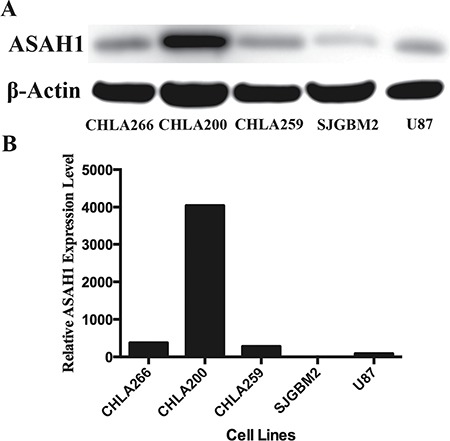
Patient derived pediatric brain tumor cells express high levels of ASAH1 (**A**) Western blot of 4 patient derived pediatric brain tumors and 1 adult glioblastoma cell line (U87) are shown. The blot was overexposed to show the faint ASAH1 band from SJGBM2 cells. (**B**) Quantitation of the relative ASAH1 expression level was performed with ImageJ. CHLA200 cells express many folds higher level of ASAH1 than other tumors.

ASAH1 has been shown to correlate with prostate tumor progression, and poor outcomes [11, 12]. To better understand the role of ASAH1 in pediatric brain tumors, the expression level of ASAH1 in these pediatric tumor cell lines were analyzed. Western blot analysis of CHLA259, CHLA266, SJGBM2, and CHLA200 cells demonstrated significant expression levels of ASAH1 (Figure 1). Of the four, the CHLA200 line, which was generated from a recurrent tumor at autopsy believed to be resisted to both chemo- and radiotherapy [13, 14], was noted to have the highest expression level of ASAH1, many fold higher (Figure 1). The SJGBM2 line, which was also generated at autopsy and had progressed despite the previous treatment with chemotherapy, contained the lowest level of ASAH1 among cell lines being studied (Figure 1). Of the four, only the CHLA200 line had prior radiation [14, 15]. CHLA259 and CHLA266 lines were generated at the time of diagnosis, prior to any therapies [14].

### Pediatric brain tumor cells are highly sensitive to ASAH1 inhibitors: OE and carmofur

**Figure 2:**
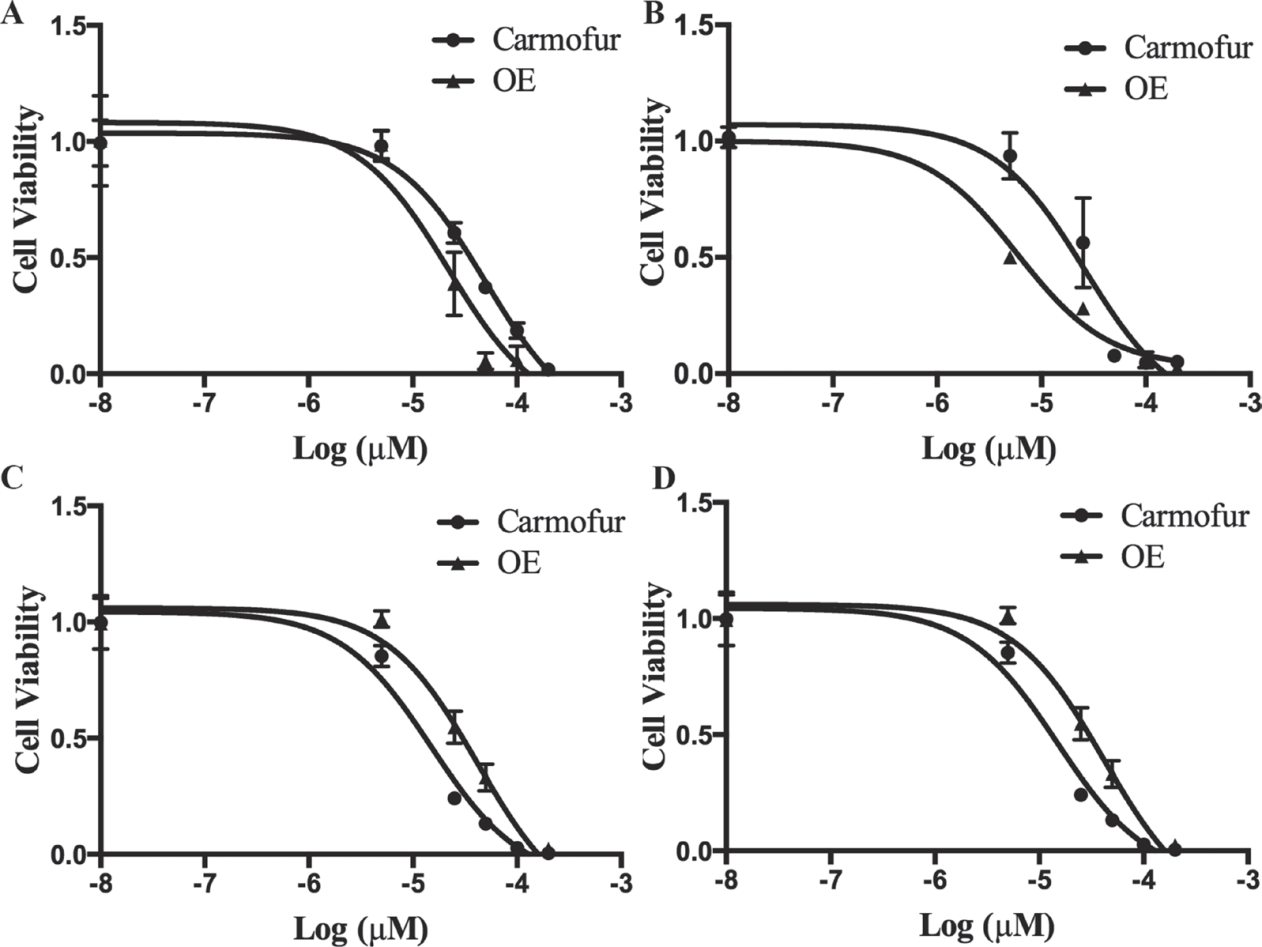
Pediatric brain tumor cells are highly sensitive to ASAH1 inhibitors: OE and carmofur MTT assays of SJGBM2 (**A**), CHLA259 (**B**), CHLA200 (**C**), and CHLA266 (**D**) were performed with carmofur and OE. Results are expressed as means and ± s.e.m (*N* = 3).

To examine the role of ASAH1 in survival of pediatric brain tumor cells, we used previously identified ASAH1 inhibitors N-oleoylethanolamine (OE) and carmofur [10, 16, 17]. To study their efficacy, MTT assays were performed with OE and carmofur against CHLA259, CHLA266, SJGBM2, and CHLA200 cells [10, 16]. Despite their known multidrug resistance phenotype, all four cell lines were highly sensitive to OE and carmofur with IC_50_ values ranging from 4.6, 50 μM (Figure 2 and Table 1). CHLA200 cells with the highest expression level of ASAH1 exhibited the most sensitivity to both OE and carmofur having IC_50_ values of 5.6 and 26 μM for OE and carmofur, respectively (Table 1).

**Table 1:** IC_50_ values range 4.5–50 μM

Cell Lines	Carmofur IC_50_	N-oleoylethanolamine IC50
SJGBM2	50 μM ± 1 μM	22 μM ± 2 μM
CHLA259	26 μM ± 2 μM	5.6 μM ± 1 μM
CHLA200	13 μM ± 3 μM	4.5 μM ± 1 μM
CHLA266	15 μM ± 1 μM	41 μM ± 2 μM

Carmofur and OE IC_50_ values from 4 different pediatric brain tumor cell lines are shown.

### Pediatric brain tumor cells treated with carmofur underwent apoptosis as demonstrated by Annexin-V-Alexa-488 conjugate staining

**Figure 3:**
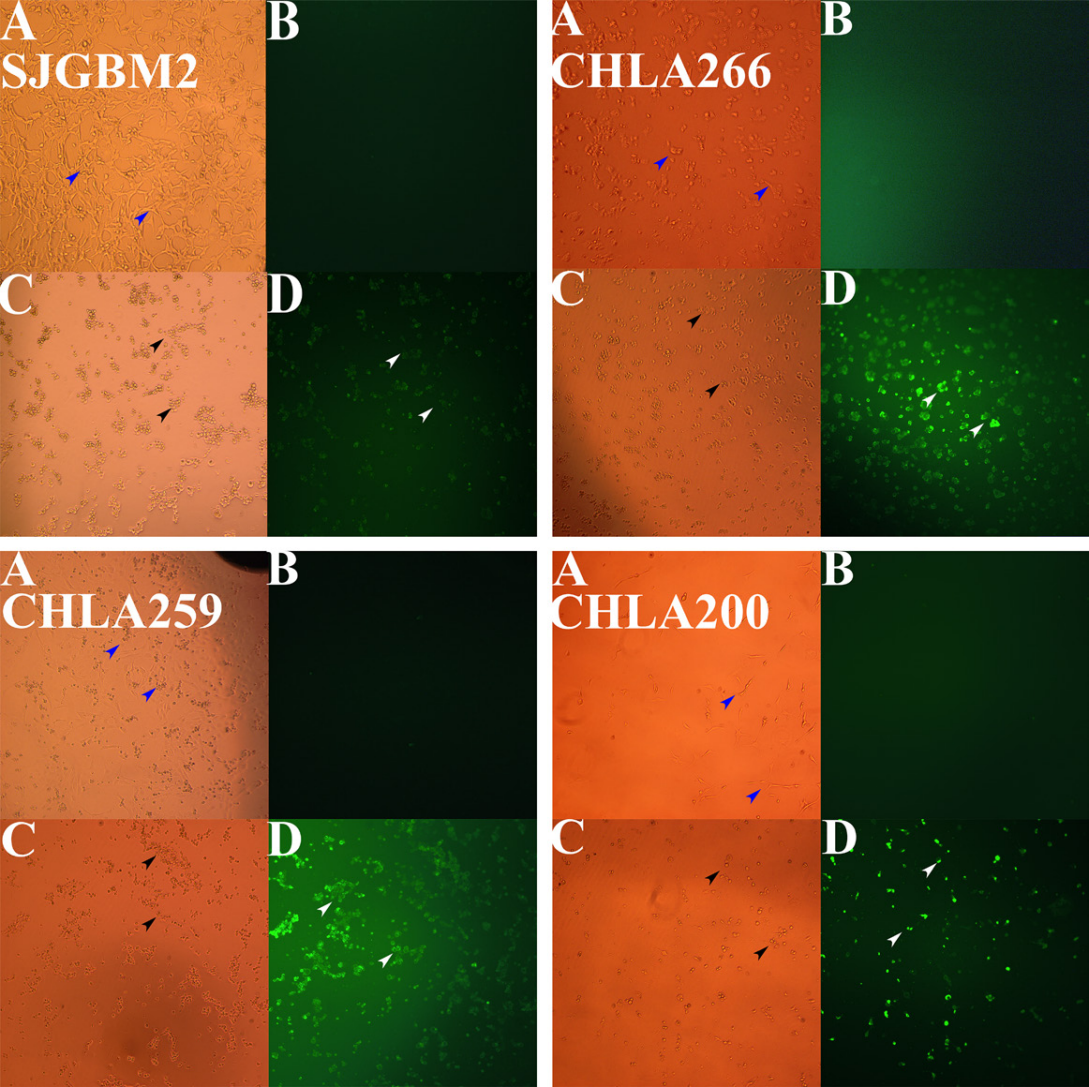
Pediatric brain tumor cells treated with carmofur underwent apoptosis as demonstrated by the Annexin-V-Alexa-488 conjugate staining Microscopy studies pediatric brain tumors are shown. Top left panel, SJGBM2; top right panel, CHLA266; bottom left panel, CHLA259; bottom right panel, CHLA200; control cells imaged with the brightlight (**A**), with the fluorescent light (**B**) vs cells treated (12 hrs) with 50 μM carmofur imaging with the brightlight (**C**), with the fluorescent light (**D**). Brightlight imaging of control live cells (blue arrows) and death cells (black arrows) are shown. Whereas a large number of apoptotic cells stained with Annexin-V-Alexa-488 (white arrows) were observed under fluorescent imaging when treated with carmofur, very little to no staining was seen in control untreated cells (B).

It has been well demonstrated that inhibition of acid ceramidase will elevate the ceramide level, which in turn induces cell apoptosis [16, 18–20]. To test this theory, tumor cells treated with carmofur were exposed to the Annexin-V-Alexa-488 conjugate to evaluate for the evidence of apoptosis. As expected, CHLA259, CHLA266, SJGBM2, and CHLA200 cells treated with carmofur underwent significant stainings with the Annexin-V-Alexa-488 conjugate as demonstrated by fluorescent signals, which strongly suggested these cells underwent apoptosis (Figure 3). Cells that were not exposed to carmofur demonstrated no significant fluorescent signals. Compared to control, cells treated with carmofur became more rounded, changing their shapes, and appeared to be in various stages in disintegration.

### Pediatric brain tumors were more sensitive to carmofur than temozolomide

**Figure 4:**
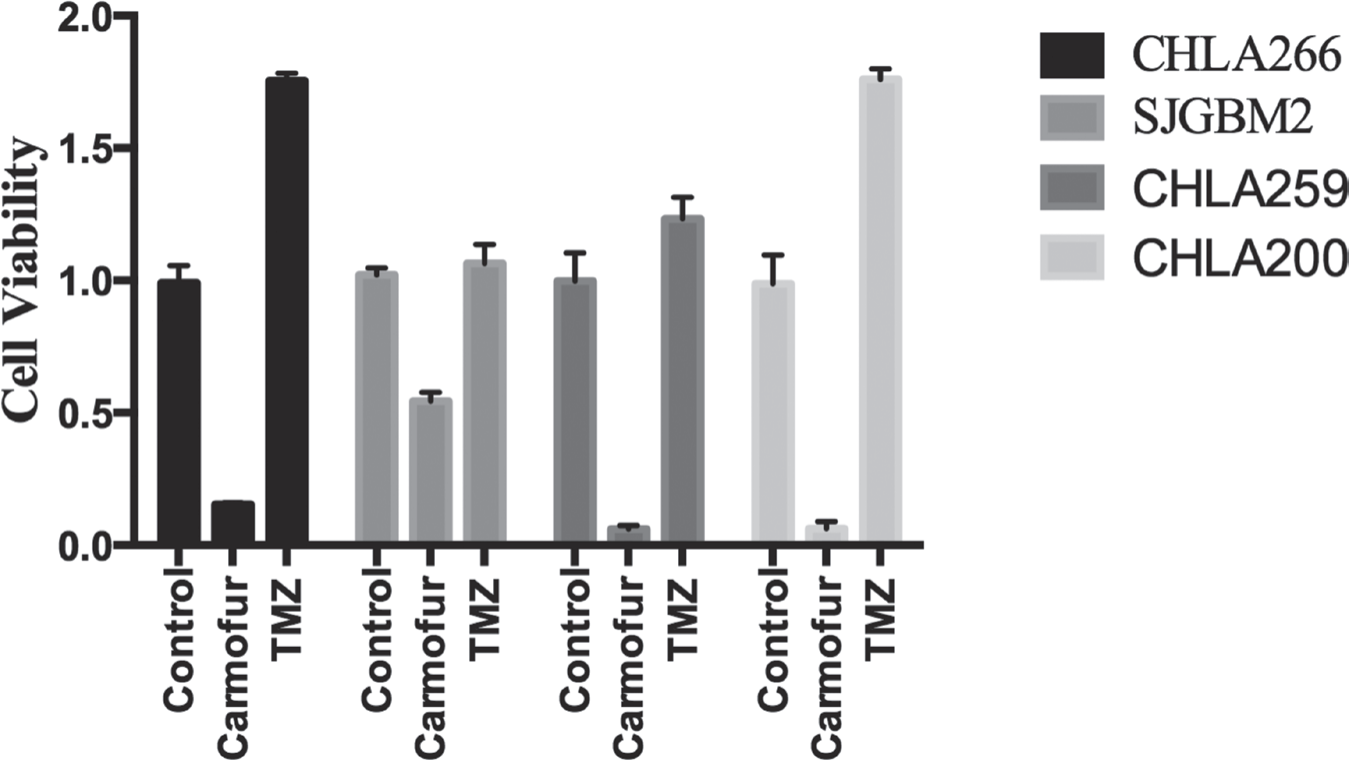
Pediatric brain tumors were more sensitive to carmofur than temozolomide Cell survival studies using MTT assays of various pediatric brain tumors are shown for control cells vs cells treated with 50 μM of carmofur and 100 μM of TMZ. Results are expressed as means and ± s.e.m (*N* = 3).

TMZ is the only chemotherapy drug that has been approved for adult glioblastoma [21]. To compare the effectiveness of carmofur and TMZ at killing tumor cells, we performed MTT cell survival studies with these drugs. MTT assays demonstrated that minimal cell death occurred in CHLA259, CHLA266, SJGBM2, and CHLA200 cells treated with TMZ, while at just half the concentration of TMZ, carmofur treatment resulted in > 50% cell death (Figure 4). Moreover, TMZ actually enhanced the cell growth of CHLA266 and CHLA200 by almost 2-folds compared to the control (Figure 4).

### A high level of ASAH1 is associated with a lower level of its substrates and a higher level of its endproducts, ceramides and sphingosines, respectively

**Figure 5:**
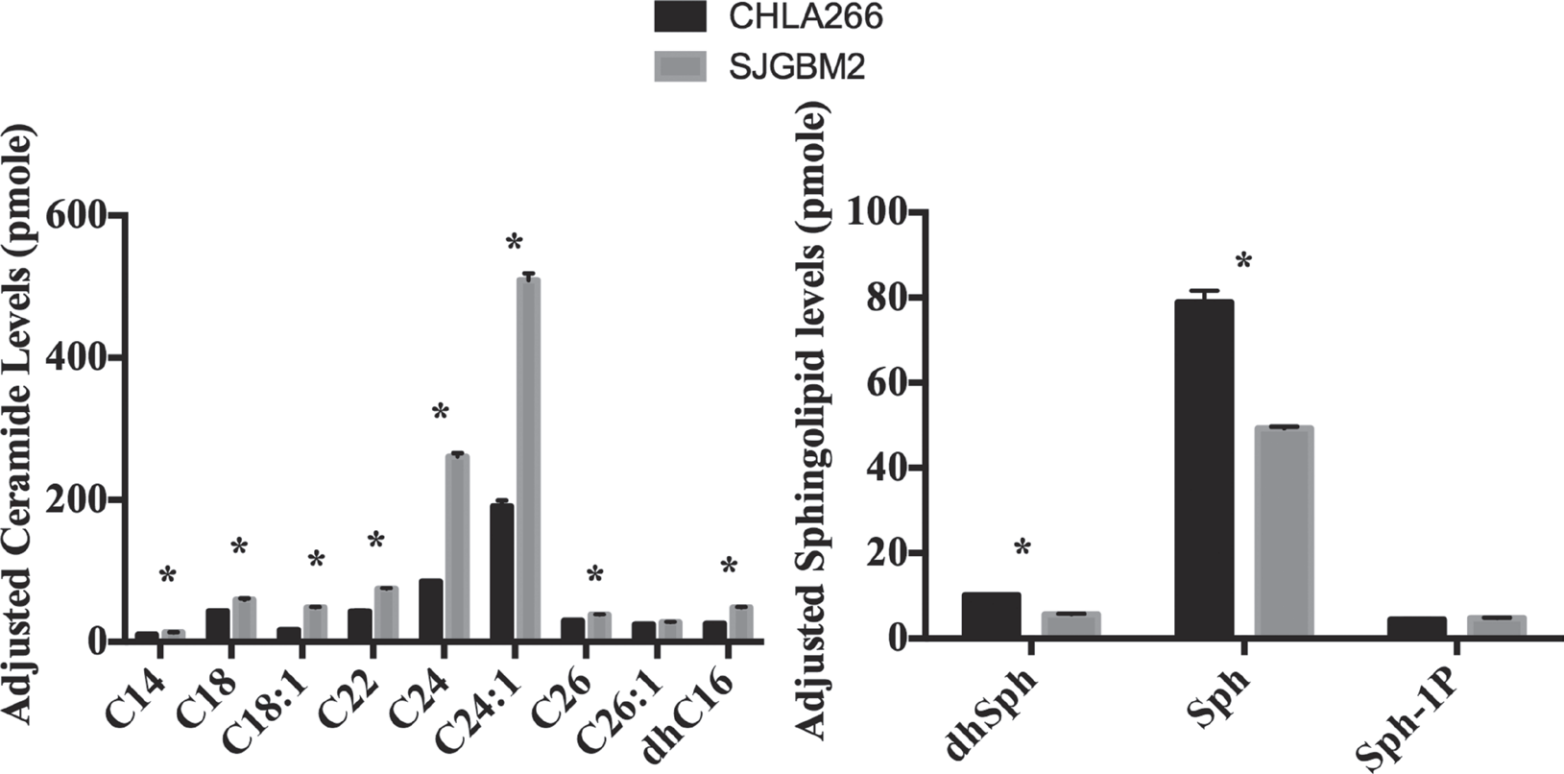
A high level of ASAH1 is associated with a lower level of its substrates and a higher level of its endproducts, ceramides and sphingosines, respectively Cell pellets were prepared and lipids were extracted for mass spectrometry as described in Materials and Methods. Intracellular ceramide and sphingolipid levels are from CHLA266 (black bars) and SJGBM2 (gray bars) cells. These levels were adjusted for equal loading based on the phosphate level. Results shown are mean ± SD of three replicates. *P < 0.05.

To determine whether a higher level of ASAH1 can modulate the sphingolipid pathway, sphingolipid levels were determined in cells with high (CHLA266) and low (SJGBM2) levels of ASAH1 (Figure 1). As expected, ceramide, the substrate of ASAH1, levels were found to be decreased in CHLA266 cells compared to SJGBM2 cells (Figure 5). In consistent with this pathway, the endproduct of ASAH1, which is sphingosine (Sph), is higher in CHLA266 cells than from SJGBM2 cells. While dihydrosphingosine (dhSph), and sphingosine (Sph) levels were increased in CHLA266 cells, the sphingosine-1-phosphate (Sph-1P) level remained unchanged between CHLA266 and SJGBM2 cells (Figure 5).

### Treatment of SJGBM2 cells with carmofur resulted in intracellular accumulation of ceramides

**Figure 6:**
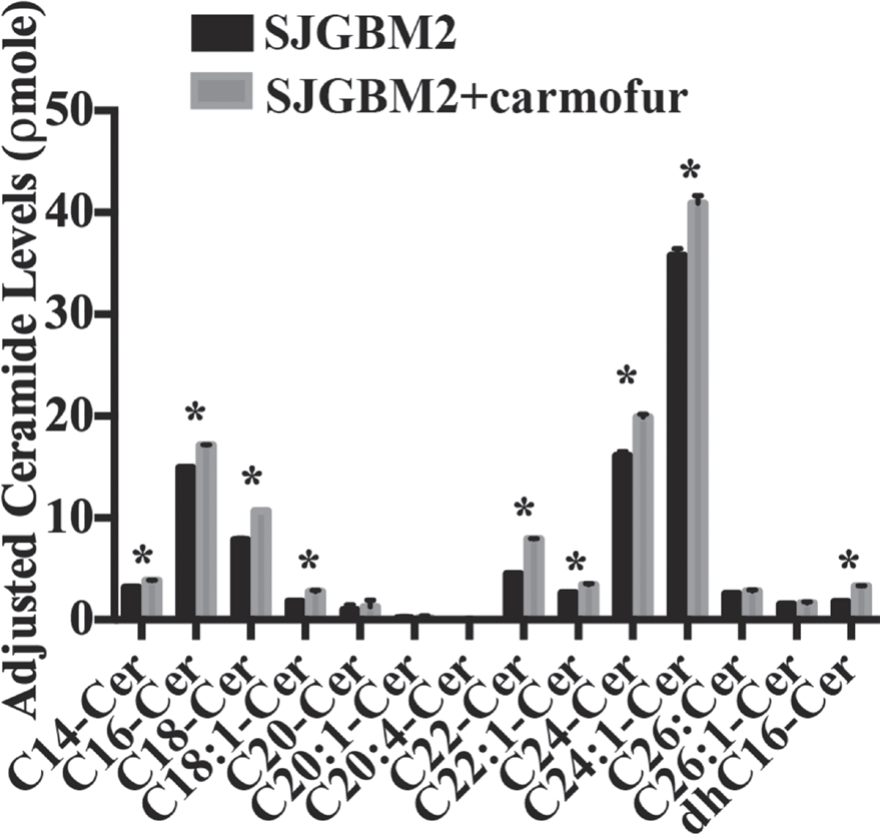
Treatment of SJGBM2 cells with carmofur resulted in intracellular accumulation of ceramides Cell pellets were prepared and lipids were extracted for mass spectrometry as described in Materials and Methods. Intracellular ceramide and sphingolipid levels are from SJGBM2 cells (black bars) and SJGBM2 cells treated with 50 μM carmofur (gray bars). These levels were adjusted for equal loading based on the phosphate level. Results shown are mean ± SD of three replicates. *P < 0.05.

In an effort to elucidate the mechanism of action, we decided to evaluate the change in intracellular level of ceramides following carmofur treatment. Treating cultures of SJGMB2 cells with 50 μM carmofur resulted in intracellular accumulation of various ceramide species, which were identified and quantified by liquid chromatography and mass spectrometry (Figure 6). This finding, which is in consistent with other study [10], suggests that carmofur inhibits ASAH1 activity and elevates tissue ceramide levels.

## DISCUSSION

**Figure 7:**
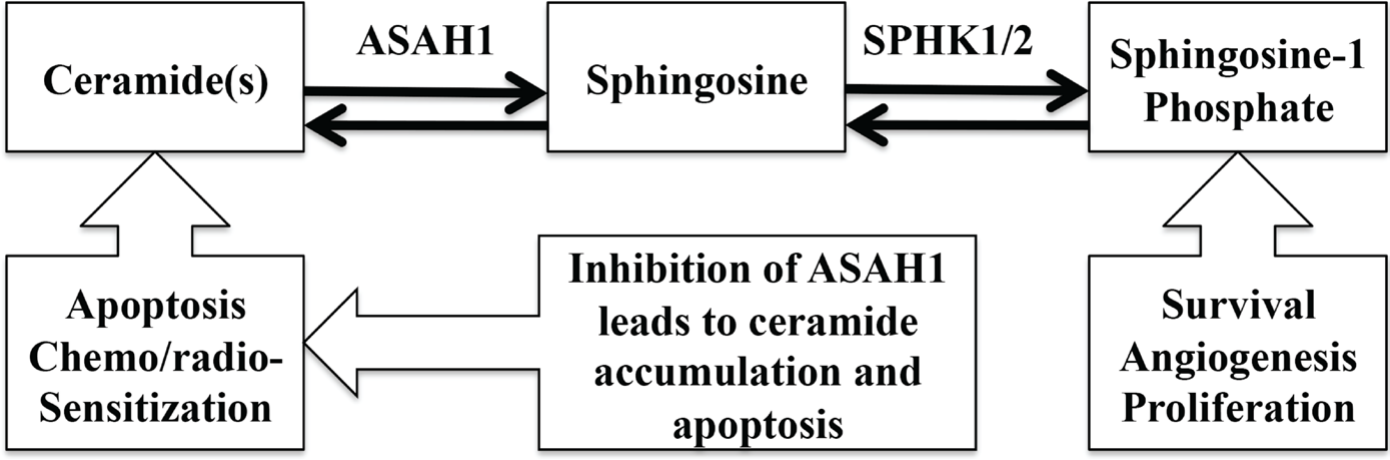
The schematic diagram of the metabolic ceramide pathway is shown ASAH1 converts ceramide into sphingosine, which is subsequently metabolized to sphingosine-1-phosphate by SPHK1 or 2. Ceramide is a tumor suppressor, promoting apoptosis and chemo/radio-sensitization. Sphingosine-1-phosphate is a tumor promoter, enhancing survival, angiogenesis, and proliferation.

ASAH1, a lysosome cysteine amidase, plays an important role in the metabolism of sphingolipids by converting ceramide into sphingosine and free fatty acid [18, 22, 23] (Figure 7). Sphingosine is phosphorylated into a tumor promoter sphingosine-1-phosphate (Sph-1P), by sphingosine kinase 1 (SPHK1) or 2 (SPHK2) (Figure 7) [22]. Sph-1P stimulates glioblastoma cell invasiveness in vitro via the up-regulation of the urokinase plasminogen activator, its receptor, and proinvasive molecule CCN1 [24, 25]. Ceramide has been shown to induce apoptosis in cells that have undergone radio- and chemotherpay [18, 22, 23, 26]. The mechanism of action of ceramide appears to relate to the release of cytochrome c leading to the activation of caspase-9 and caspase-3 [17]. ASAH1 is associated with tumor progression and invasiveness especially in melanoma, colon, and prostate cancers [11, 12, 27]. In particular, the treatment of the adult glioblastoma U87MG cell line with OE sensitizes these cells to radiation [16]. Among cell lines being studied, our Western blot data revealed the highest expression level of ASAH1 in CHLA200 cells (Figure 1). CHLA200 was generated at autopsy from a recurrent tumor invading the hemisphere, brainstem, and cerebellum, which had been previously treated with vincristine and radiotherapy [14]. Despite the prior radiation and chemotherapy, CHLA200 tumors still recurred in the patient that the cell line was derived from, which suggests this tumor is both radio- and chemoresistant [13, 14]. Consistent with this finding, other investigators reported: 1) that over-expressed ASAH1 in prostate cells led to larger tumor volumes that are more resistant to chemotherapy; 2) when ASAH1 is suppressed, cells become more sensitive to chemotherapy; and 3) the treatment with B13, an ASAH1 inhibitor, sensitizes these cells to radiation [11, 28]. We speculate that the high expression level of ASAH1, that was either induced by radiation or naturally occurring in the subpopulation of cells, enables CHLA200 cells to survive the prior radio- and chemotherapy. Due to the ability of ASAH1 to metabolize ceramides into Sph, we also found that cells containing a higher level of ASAH1 are also associated with a lower level of ceramides and a higher level of Sph. Not too unexpectedly is that the intracellular level of Sph-1P is not influenced by the level of ASAH1 as other researchers have been shown that Sph-1P can be secreted into the extracellular space allowing cells to maintain a steady level of intracellular Sph-1P [23–25, 29] (Figure 5). Given its important role in cancer formation, prior studies have suggested ASAH1 as a novel anti-cancer target in other non-central-nervous-system cancers [20]. To test the effectiveness of ASAH1 inhibitors at targeting pediatric brain tumors, we treated these pediatric brain tumor cell lines with known ASAH1 inhibitors, carmofur and OE [10, 16, 30]. Despite their multidrug resistance phenotype, CHLA200, SJGBM2, CHLA266, and CHLA200 cells remain highly sensitive to carmofur and OE [13, 14] (Figure 2 and Table 1). No clear correlation between IC_50_ values and the level of ASAH1 expression was observed. This could be a result of having 4 different unrelated cells lines. In addition to the difference in ASAH1 expression level, the difference in the condition that the drug will be presented with in each of these cell lines could also influence IC_50_ values. Cells treated with ASAH1 inhibitors are induced to undergo apoptosis due to the elevated level of ceramide (Figure 6) [18, 22]. As expected, we demonstrated that all of these cell lines underwent apoptosis when treated with carmofur as evidenced by the binding of the Annexin-V-Alexa-488 conjugate, which produced the fluorescent signal observed under fluorescent microscopy (Figure 3). For comparison, we evaluated the effectiveness of TMZ against these pediatric brain tumor cell lines. Not surprisingly, given their known multidrug resistance phenotype, TMZ was not effective at targeting any of the pediatric brain tumors tested (Figure 4). For unclear reason(s), TMZ actually enhanced the cell growth of CHLA200 and CHLA266 cell lines when treated over a period of 12 hours. A recent report by Stepanenko et al demonstrated that TMZ treatment of glioblastoma cells results in diverse responses that include enhanced proliferation and migration due to chromosomal instability induced by TMZ [31]. Further investigation is needed to determine if a similar mechanism of effect is playing a role here as well. These results suggest that carmofur is apt to be much more effective than TMZ and is a promising drug that should be further evaluated to treat multiple pediatric brain tumors. Carmofur has been used as a treatment for colorectal cancers since 1981 and has been shown to be able to cross the blood-brain barrier [10, 30].

## MATERIALS AND METHODS

### Reagents and cells

Mouse antibody against ASAH1 (612302) was purchased from BD Biosciences (San Jose, CA). Anti-actin, carmofur, temozolomide (TMZ), and N-oleoylethanolamine (OE), 3-(4,5-dimethylthiazol-2-yl)-2,5-diphenyltetrazolium bromide (MTT) were purchased from Sigma Aldrich (St. Louis, MO). DMSO, used to dissolve carmofur, temozolomide, and OE, was purchased from Sigma Aldrich (St. Louis, MO). HRP-conjugated goat anti-mouse IgG was supplied by R&D Systems, Inc. (Minneapolis, MN). SDS-PAGE and Western blotting materials and the Annexin V kit were obtained from Life Technologies, Inc. (Grand Island, NY).

### Cells

Pediatric Anaplastic medulloblastoma (CHLA259), pediatric glioblastoma (SJGBM2 and CHLA200), and pediatric atypical teratoid rhabdoid tumors (CHLA266) were obtained from the Children's Oncology Group (COG) Cell Culture and Xenograft Repository [13–15]. SJGBM2 and CHLA200 are classified as glioblastoma, not otherwise specified (NOS), CHLA259 as medulloblastoma, NOS, according to the World Health Organization (WHO) 2016 classification of tumors of the central nervous system [32]. These cells were grown in Iscove's modified Dulbecco's medium supplemented with 20% fetal bovine serum, 4mM L-glutamine, and 1X ITS (5 μg/mL insulin, 5 μg/mL transferrin, 5 ng/mL selenous acid).

### Western blot analysis and quantification

Equal amounts (15μg) of protein from each of the tumor samples were loaded onto the 4, 12% gel. SDS-PAGE and Western blotting were performed using standard methods. Gel is blocked with 5% bovine serum albumin. A 1:500 dilution was used for primary antibody and 1:10,000 for secondary antibody. ImageJ software was used to quantify Western blot images.

### 3-(4,5-dimethylthiazol-2-yl)-2,5-diphenyltetrazolium bromide (MTT) assays

Cells were plated onto a 96-well plate at the density of 1 × 10^5^ cells/ml. Media was exchanged to serum-free media after overnight incubation. Cells were treated with various drugs (OE, carmofur) dissolved in DMSO for 24 hours. MTT reagents were added after 24 hours of incubation, followed by acidic-isopropanol 4 hours later to dissolve formazan. All wells contained less than 0.2% of final concentration of DMSO. The absorbance values were recorded at wavelengths 570 and 630 nm. IC_50_'s were calculated with the GraphPad Prism software.

### Sphingolipid quantification

Electrospray ionization tandem mass spectrometry (ESI/MS/MS) analysis of endogenous (phyto)sphingosine bases, sphingoid base-1-phosphates and (phyto)ceramide species were performed on a Thermo Fisher Quantum triple quadrupole mass spectrometer, operating in a Multiple Reaction Monitoring (MRM) positive ionization mode, using the modified version [33]. Briefly, biological materials were fortified with the internal standards (ISs: C_17_ base D-erythro-sphingosine (17CSph), C_17_ sphingosine-1-phosphate (17CSph-1P), N-palmitoyl-D-erythro-C_13_ sphingosine (13C16-Cer) and heptadecanoyl-D-erythro-sphingosine (C17-Cer) and C6-Phyto-ceramide), then extracted with ethyl acetate/iso-propanol/water (60/30/10 %v/v) solvent system. After evaporation and reconstitution in 150 μl of methanol, samples were injected on the HP1100/TSQ Quantum LC/MS system and gradient eluted from the BDS Hypersil C8, 150 × 3.2 mm, 3 μm particle size column, with 1.0 mM methanolic ammonium formate/2 mM aqueous ammonium formate mobile phase system. Peaks corresponding to the target analytes and internal standards were collected and processed using the Xcalibur software system. Quantitative analysis was based on the calibration curves generated by spiking an artificial matrix with the known amounts of the target analyte synthetic standards and an equal amount of the internal standards (ISs). The target analyte/IS peak areas ratios were plotted against analyte concentration. The target analyte/IS peak area ratios from the samples were similarly normalized to their respective ISs and compared to the calibration curves, using a linear regression model. Introduction of the internal standards to the samples prior to extraction, yields results already “recovery corrected,” therefore, no further data manipulation are necessary.

### Fluorescent microscopy

Cells were plated onto a 96-well plate at the density of 1 × 10^4^ cells/ml. Media was exchanged to serum-free media after overnight incubation. Cells were treated with various drugs for 12 hours then labeled with the Annexin-V-Alexa-488 conjugate according to the manufacturer's instructions. Imaging was recorded with a Zeiss Axiovert 200M inverted fluorescent microscope.

## CONCLUSIONS

We demonstrated that medulloblastoma, pediatric glioblastoma, and atypical teratoid rhabdoid cell lines express significant levels of acid ceramidase, where levels are highest in the radioresistant tumors, suggesting that acid ceramidase may confer radioresistance. More importantly, we also showed that acid ceramidase inhibitors are highly effective at targeting these pediatric brain tumors with low IC_50_ values (4.6, 50 μM). This data suggests acid ceramidase could be used as a novel drug target for adjuvant pediatric brain tumor therapies. Of these acid ceramidase inhibitors, carmofur has seen clinical use in Japan since 1981 for colorectal cancers and is a promising drug to undergo further animal studies and subsequently a clinical trial for pediatric patients with brain tumors.

## Abbreviations

ASAH1: acid ceramidase
ATRT: atypical teratoid rhabdoid tumors
DMSO: dimethyl sulfoxide
ESI/MS/MS: electrospray ionization tandem mass spectrometry
GBM: glioblastoma
dhSph: dihydrosphingosine
IC_50_: half maximal inhibitory concentration
ISs: internal standards
MTT: 3-(4,5-dimethylthiazol-2-yl)-2,5-diphenyltetrazolium bromide
OE: *N*-oleoylethanolamine
SDS-PAGE: sodium dodecyl sulfate polyacrylamide gel electrophoresis
Sph: sphingosine
Sph-1P: sphingosine-1-phosphate
TMZ: temozolomide
